# CAR-NK Cell Therapy: A Transformative Approach to Overcoming Oncological Challenges

**DOI:** 10.3390/biom14081035

**Published:** 2024-08-20

**Authors:** Wangshu Li, Xiuying Wang, Xu Zhang, Aziz ur Rehman Aziz, Daqing Wang

**Affiliations:** 1 China Key Laboratory for Early Diagnosis and Biotherapy of Malignant Tumors in Children and Women, Dalian Women and Children’s Medical Group, Dalian 116012, China; doctor_lws@outlook.com (W.L.); 18098857006@163.com (X.W.); 2 Department of Obstetrics and Gynecology, Shengjing Hospital of China Medical University, Shenyang 110004, China; 3 The Second Affiliated Hospital of Harbin Medical University, Harbin 151801, China; zhangxu@hrbmu.edu.cn

**Keywords:** CAR-NK cell therapy, Immunotherapy, tumor heterogeneity

## Abstract

The use of chimeric antigen receptor (CAR) in natural killer (NK) cells for cancer therapy is gaining momentum, marking a significant shift in cancer treatment. This review aims to explore the potential of CAR-NK cell therapy in cancer immunotherapy, providing a fresh perspective. It discusses the innovative approaches in CAR-NK cell design and engineering, particularly targeting refractory or recurrent cancers. By comparing CAR-NK cells with traditional therapies, the review highlights their unique ability to tackle tumor heterogeneity and immune system suppression. Additionally, it explains how novel cytokines and receptors can enhance CAR-NK cell efficacy, specificity, and functionality. This review underscores the advantages of CAR-NK cells, including reduced toxicity, lower cost, and broader accessibility compared to CAR-T cells, along with their potential in treating both blood cancers and solid tumors.

## 1. Introduction

The inception of chimeric antigen receptor (CAR) technology has revolutionized the landscape of cancer therapy. Originating from its development for T cells in the late 20th century, CAR therapy has demonstrated profound efficacy, particularly in treating hematologic malignancies [1]. CARs, engineered to augment T cell recognition and the eradication of tumor cells, amalgamate external antigen recognition domains with internal signaling domains, thereby bestowing CAR-T cells with tumor-specific targeting capabilities [2]. However, despite the progress, the application of CAR-T therapy in treating solid tumors remains fraught with challenges, including severe side effects like cytokine release syndrome (CRS), targeting precision issues, and the emergence of drug resistance [3].

Natural killer (NK) cells are essential components of the innate immune system. Unlike T cells, which recognize peptides presented by major histocompatibility complex (MHC) molecules, NK cells display receptors that recognize stress-induced autologous proteins on cancer cells. Their functional activity is inhibited by MHC molecules displayed on such cells [4]. Their distinct attribute, along with their unique cytokine profile, positions CAR-NK cell therapy as an innovative alternative, potentially circumventing the limitations of CAR-T therapy. The adaptation of CAR technology to NK cells has opened new avenues for cancer treatment, particularly in addressing the complexities of solid tumors, tumor heterogeneity, and immune escape mechanisms [5].

This review delves into the progression, advantages, and challenges of CAR-NK cell therapy, with a keen focus on its potential to overcome the hurdles in treating solid tumors. We will systematically evaluate the efficacy of CAR-NK cells in enhancing cancer treatment, their role in circumventing drug resistance, and their capacity to minimize toxic reactions. By providing a comprehensive overview of the state of the art in CAR-NK therapy, this paper seeks to address the scientific queries and literature gaps within this field. Through a detailed analysis of the development, benefits, and obstacles associated with CAR-NK cell therapy, this review aims to chart the future course of this promising treatment modality. We aspire to offer a current perspective on CAR-NK cell therapy, elucidating its evolving role and prospects in the advancing frontier of cancer immunotherapy.

## 2. Overview of CAR-NK Cell Therapy

CAR-NK cell therapy represents a burgeoning area within cancer immunotherapy, entailing the genetic engineering of NK cells to express CARs. These receptors are meticulously designed to include an extracellular antigen-binding domain, commonly sourced from a monoclonal antibody, fused with an intracellular signaling domain. This domain typically features CD3ζ and may be augmented with one or more co-stimulatory molecules, such as CD28 or 4-1BB, to enhance the therapeutic efficacy and functionality of the CAR-NK cells [6]. NK cells, crucial for immunotherapy against cancer, recognize stressed cells independently of neoantigen presentation. They exhibit enhanced activity against tumors that lack MHC class I expression due to acquired resistance mechanisms. Researchers are exploring ways to mobilize endogenous NK cells or provide ex vivo-expanded NK cells as cellular therapy. Such a capability represents a significant advantage, distinguishing CAR-NK cells from other immunotherapeutic approaches [7].

**Figure 1:**
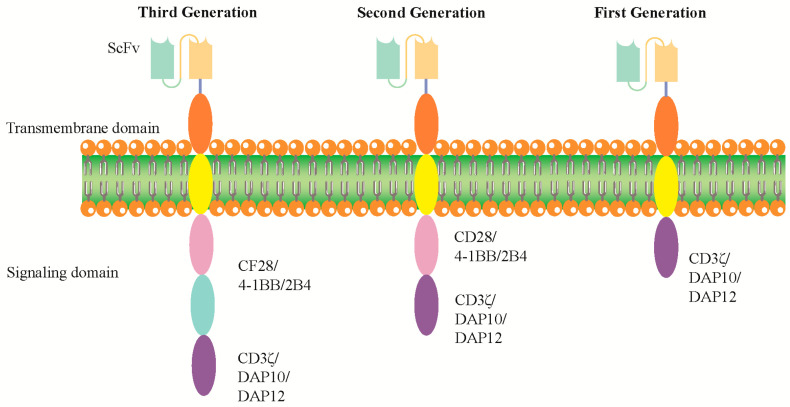
Specific CAR constructs for CAR-NK cells. The extracellular domain is constant; the single-chain variable fragment (scFv) sequence changes depending on the antigen. The first generation has an intracellular tyrosine-based activation motif, such as a CD3 ζ chain, DAP10 or DAP12. Second and third generations have one and two co-stimulatory domains (CD28, 4-1BB or 2B4), respectively [16,17].

The flexibility of CAR-NK cell therapy is multifaceted, showcasing both the diversity in the cellular sources and the molecular intricacy of the CAR constructs themselves. Initially, this adaptability is evident in a variety of sources from which CAR-NK cells can be derived, including peripheral blood, umbilical cord blood, induced pluripotent stem cells (iPSCs), and NK cell lines (reviewed in [8,9]). Each source offers unique advantages in terms of availability, expansion potential, and compatibility with genetic modification. Building upon this foundational versatility, Figure 1 highlights the specific molecular engineering of CAR constructs that further expands the therapeutic potential of CAR-NK cells. It provides some effective CARs that have been published, detailing their molecular combinations of extracellular and intracellular domains, alongside their respective target antigens. This precision in design allows for the creation of CAR-NK cells tailored to recognize and eliminate a wide array of cancer cell antigens with remarkable specificity. Among these, the NK-92 cell line stands out due to its high cytotoxic potential and ease of expansion in vitro. The NK-92 cells, once modified to express the CAR, become potent tools in targeting and destroying tumor cells [10]. A crucial aspect of CAR-NK cell therapy is its potential to be used as an “off-the-shelf” product, offering versatile and readily available treatment options. This characteristic is particularly beneficial in the clinical setting as it circumvents the need for patient-specific matching or lymphodepletion procedures (reviewed in [11,12]). It significantly simplifies the treatment process, potentially making it accessible to a broader patient population and reducing time and costs associated with therapy customization. Moreover, the manufacturing of CAR-NK cells involves a series of sophisticated steps to ensure their safety, efficacy, and consistency [13]. This includes the selection of the right target antigen, optimization of CAR design for maximum binding and activation, and fine-tuning the cultivation process to produce cells with high vitality and functional stability. In addition to their direct cytotoxicity, CAR-NK cells also exhibit an array of immunomodulatory functions [14]. They can recruit other immune cells to the tumor site, enhancing the overall anti-tumor response. This synergy between direct tumor cell killing and immune system activation is a key factor that could potentially increase the effectiveness of CAR-NK cell therapy in treating a wide range of cancers.

Furthermore, the field of CAR-NK cell therapy is rapidly evolving, particularly when compared to established CAR-T cell therapies. Researchers are keenly focused on addressing certain limitations that are more pronounced in the CAR-NK cell domain [15]. These include challenges such as the limited persistence of CAR-NK cells in vivo, a concern less prevalent in CAR-T cells due to their longer-lasting memory and proliferation capabilities. Additionally, potential off-target effects and the immunosuppressive nature of the tumor microenvironment are areas where CAR-NK cells, much like their CAR-T counterparts, require further refinement and innovation [3].

## 3. The Comparison of CAR-NK Cell and CAR-T Cell Therapies

CAR-NK and CAR-T therapies are powerful tools against cancers, originating primarily from research in hematological malignancies (blood cancers). However, there exist several similarities and differences between them. Below is a brief comparison of both therapies (Table 1).

### 3.1. Intracellular Signaling Domains

CAR-T cells are typically engineered to incorporate the CD3ζ domain along with co-stimulatory domains such as CD28 and 4-1BB [18]. The CD3ζ domain plays a pivotal role in signal transduction and is an essential component of the T cell receptor (TCR), linking antigen recognition to several intracellular signal-transduction pathways. The co-stimulatory domains, CD28 and 4-1BB, enhance the survival, proliferation, and effector functions of the CAR-T cells [19].

CAR-NK cells can utilize NK-specific signaling domains like 2B4, DAP10, and DAP12. These NK-specific signaling domains contribute to the unique functional characteristics of CAR-NK cells. For instance, the 2B4 domain serves as a co-stimulatory domain, enhancing the cytotoxic activity of CAR-NK cells [20]. DAP10, a major adaptor protein and the exclusive signaling intermediate of NKG2D in human NK cells, is known to enhance the cytotoxic ability of CAR-NK cells against certain tumor cells [21]. DAP12, an important adaptor molecule that is associated with multiple activating receptors, is also utilized in CAR-NK cells to improve their anti-tumor potential [22].

**Figure 2:**
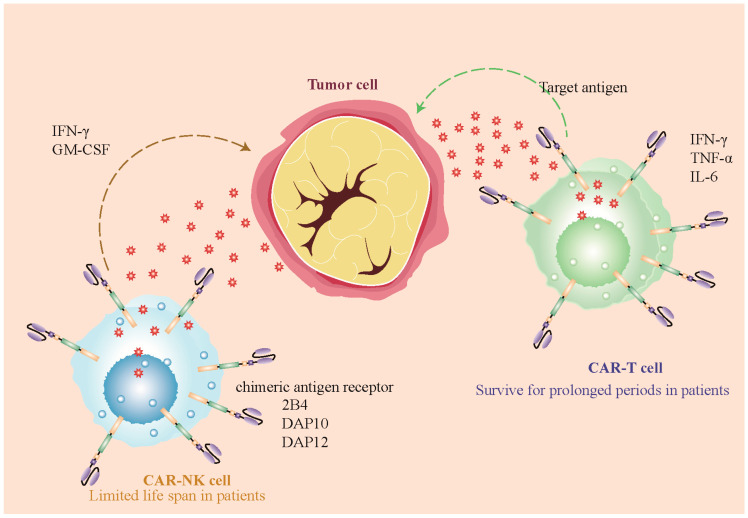
CAR-T and CAR-NK cell therapy mechanisms against tumor cells. The left side of the image represents CAR-NK cells, which recognize target antigens on the surface of tumor cells through CAR. These cells have 2B4 and DAP10/DAP12 as activating ligands and can secrete cytokines such as IFN-γ and GM-CSF. The right side represents CAR-T cells, which also recognize and bind to target antigens on tumor cells through the chimeric antigen receptor. CAR-T cells signal molecules such as CD3ζ, CD28, and 4-1BB and can secrete cytokines like IFN-γ, TNF-α, and IL-6; the tumor cells in the center show their target antigens, which are the targets for recognition and binding by CAR-T and CAR-NK cells [16,28].

Thus, both CAR-T cells and CAR-NK cells exhibit distinct differences in their intracellular signaling domains. These differences contribute to their unique functionalities in targeting and eliminating cancer cells (Figure 2).

### 3.2. Cell Sources for Both CAR-T and CAR-NK Cells

T cells are harvested from the patient, engineered to express a specific CAR, and then reintroduced into the patient [23]. Alternatively, CAR-T cells can be obtained from a donor with a compatible MHC, referred to as MHC-matched allogeneic derivation [24]. MHC comprises a group of cell surface proteins crucial for the adaptive immune system to recognize foreign entities. Ensuring MHC compatibility between the donor and the recipient is vital to avert graft-versus-host disease (GVHD).

On the other hand, CAR-NK cells can be derived from a variety of sources [12]. It has been observed that donor-versus-recipient NK cell alloreactivity could eliminate leukemia relapse and GVHD [25]. NK cells have a unique ability to target cancer cells without prior sensitization, unlike T cells. They can be transferred between individuals without strict HLA matching. Researchers are exploring various sources of therapeutic NK cells, including donor-derived NK cells and stem-cell-derived NK cells. However, not all NK cells have the same antitumor potential. Identifying and using highly functional NK cells is crucial for successful adoptive NK cell therapies. Some selection methods for NK-cell-dependent immunotherapies have been reviewed in [26]. Their ability to act in an antigen-independent manner makes them a viable option for an “off-the-shelf” therapy that can be manufactured on a large scale and easily distributed to cancer patients [12]. Moreover, NK cells from allogeneic sources can also be used in immunotherapies owing to their reduced risk of alloreactivity [27]. This means that they are less likely to attack the patient’s own cells, reducing the risk of complications (Figure 2).

So, both CAR-T and CAR-NK cells have their unique sources and advantages; the choice between them depends on various factors including the type of cancer, the patient’s condition, and the available resources. Both represent promising advances in the field of cancer immunotherapy.

### 3.3. CAR-T and CAR-NK Cells In Vitro Expansion

The expansion of CAR-T cells in vitro is essential to obtain enough effector cells for therapeutic purposes [29]. This process typically involves the activation and proliferation of T cells under controlled laboratory conditions. Initially, T cells are activated using specific stimuli, such as anti-CD3 and anti-CD28 antibodies [30]. Following the activation, the culture is supplemented with cytokines, particularly interleukin-2 (IL-2), to support the growth and proliferation of T cells [31].

Like CAR-T cells, CAR-NK cells are engineered to target and eliminate cancer cells. However, NK cells represent a different lineage of lymphocytes with innate immune properties. The in vitro expansion of CAR-NK cells can be achieved using various methods, including feeder cell lines or a combination of cytokines like IL-2 and IL-15. IL-15 is particularly significant for NK cell growth, as it promotes the survival, proliferation, and activation of NK cells [32]. The feeder cells, often genetically modified to express certain ligands or to secrete growth factors, provide a nurturing environment for CAR-NK cell expansion [33]. These feeder cells can interact with NK cell receptors, delivering necessary signals that promote cell growth and functional maturation.

**Figure 3:**
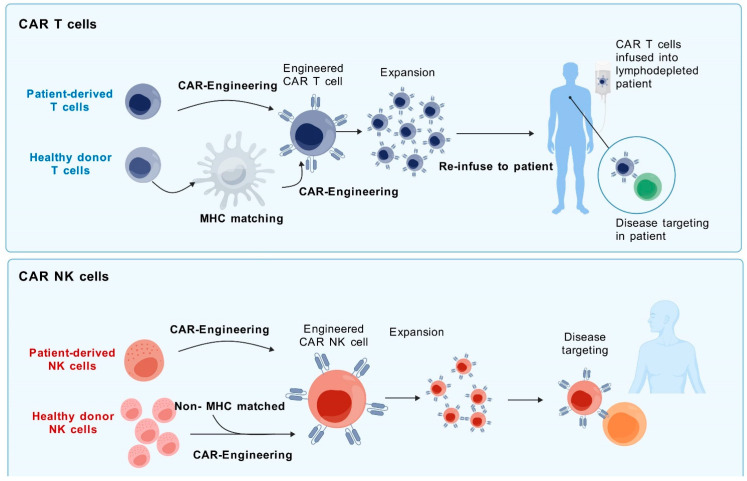
Comparison of CAR-T cell and CAR-NK cell therapy processes. The figure illustrates the entire process from cell acquisition to disease targeting for both CAR-T and CAR-NK cell therapies. For CAR-T cells, MHC matching is required to prevent rejection. For CAR-NK cells, there is less need for MHC matching [25,26], simplifying the preparation process. Both undergo engineering and expansion and are reintroduced into the patient to combat disease.

The in vitro expansion of CAR-T and CAR-NK cells is a crucial step in preparing these cells for therapeutic use in cancer immunotherapy. For in vitro expansion of both types of CAR cells, this process involves a combination of cell activation, growth factor supplementation, and sometimes the use of feeder cells to produce many effector cells capable of targeting and destroying cancer cells (Figure 3).

### 3.4. Pre-Expansion Prior to Transduction in CAR-T and CAR-NK Cells

For CAR-T cells, the pre-expansion process often involves the use of specific stimuli to activate the cells and promote their proliferation [34]. One study showed that functional CAR-T cells could be generated within 24 h from T cells derived from peripheral blood without the need for T cell activation or ex vivo expansion [35]. Another study described a 9-day protocol for CAR gene transduction and expansion of primary rhesus macaque peripheral blood mononuclear cells (PBMCs) [36]. The cells produced and expanded with this method showed high levels of viability, high levels of co-expression of two transduced genes, retention of the central memory phenotype, and enough for immunotherapeutic infusion [36].

For CAR-NK cells, the pre-expansion process is similar but has some unique aspects. One protocol described how to generate CAR-NK cells with transduction efficiencies greater than 15% from healthy donor ex vivo expanded NK cells using third-generation lentiviral vectors [37]. The protocol also showed how to assess CAR-NK cell anti-tumor function in vitro using a flow-cytometry-based killing assay [37]. Another study developed a highly efficient method for site-specific gene insertion in NK cells using CRISPR (Cas9/RNP) and AAVs [38]. The CAR transduction was efficient, its expression remained stable after expansion, and it improved efficacy against AML targets [38].

Pre-expansion prior to transduction is a critical step in the production of both CAR-T and CAR-NK cells. This process involves the stimulation and expansion of T or NK cells before they are genetically modified to express the CAR. The goal of this step is to increase the number of cells available for transduction, which can enhance the efficiency of the transduction process.

### 3.5. Cell Killing Mechanism in CAR-T and CAR-NK Therapies

CAR-T cells are engineered with CARs that specifically target and bind to antigens presented on the surface of tumor cells [39]. The external domain of these receptors, typically derived from the single-chain variable fragment (scFv) of an antibody, enables precise recognition of the antigens. Upon engaging with its target antigen, the CAR initiates a series of intracellular signals through domains such as CD3ζ and costimulatory domains like CD28 or 4-1BB [40]. Once activated, CAR-T cells deploy cytotoxic molecules, including perforin and granzymes [41].

In addition to their CAR-dependent capabilities, NK cells exhibit CAR-independent cytotoxicity, a crucial mechanism for targeting cancer cells. NK cells possess natural activating and inhibitory receptors that respond to stress signals and the absence of normal “self” markers on potential target cells [7]. The interplay of these signals determines the activation state of NK cells. Once activated, NK cells unleash cytotoxic granules, containing molecules like perforin and granzymes, which facilitate the direct killing of target cells, mirroring the cytotoxic approach of CAR-T cells [42]. This dual-action mechanism endows CAR-NK cells with a unique advantage, enabling them to effectively eradicate cancer cells, even those lacking the specific antigen recognized by the CAR [11]. This capability renders CAR-NK cells exceptionally effective and versatile in combating diverse tumor cell populations, enhancing their potential as a formidable tool in cancer immunotherapy [27].

The above data show that CAR-T cells primarily rely on antigen-specific recognition for cytotoxicity, while CAR-NK cells can kill tumor cells through both CAR-dependent and independent mechanisms, offering a broader range of targets and potentially enhancing the effectiveness of cancer immunotherapy.

### 3.6. Cytokine Release Syndrome and Neurotoxicity in CAR-T and CAR-NK Therapies

CRS and neurotoxicity are notable side effects associated with CAR-T cell therapy, presenting significant clinical challenges. CRS is an acute systemic inflammatory response triggered by the massive release of cytokines from activated CAR-T cells [43].

On the other hand, the incidence and severity of these side effects are notably lower in CAR-NK cell therapies, which might be attributed to the inherent differences in the activation pathways and effector functions of NK cells compared to T cells [7]. Additionally, the engineering aspects of CAR constructs in NK cells, including the signaling domains and co-stimulatory molecules, might also contribute to the reduced toxicity profile [27].

### 3.7. CAR-T and CAR-NK Therapies in Hematological Malignancies and Solid Tumors

CAR-T therapy has demonstrated significant efficacy in treating hematological malignancies, particularly B-cell leukemias and lymphomas, by targeting specific antigens like CD19 [44]. However, its application in solid tumors faces challenges due to issues like identifying tumor-specific antigens, T cell infiltration, and overcoming the immunosuppressive tumor microenvironment [45]. Conversely, CAR-NK therapy, which is emerging as a promising alternative, shows potential in both hematological malignancies and solid tumors [45]. CAR-NK cells can mediate their effects through both CAR-dependent and -independent mechanisms, potentially offering broader tumor recognition and less susceptibility to the immunosuppressive effects of the tumor microenvironment [11]. Moreover, CAR-NK cells have been associated with fewer severe side effects, such as cytokine release syndrome and neurotoxicity, compared to CAR-T cells, highlighting their potential for a safer profile in cancer immunotherapy [14].

Thus, CAR-T cells have established a strong foothold in treating hematological cancers, their role in solid tumor therapy remains under intensive research to overcome existing limitations [46]. CAR-NK cells, on the other hand, represent a versatile and potentially safer option, with ongoing research indicating their broader applicability and effectiveness in treating both hematological malignancies and solid tumors [47].

### 3.8. Cost-Effectiveness in CAR-T and CAR-NK Therapies

CAR-NK cells have several advantages over CAR-T cells that make them potentially more cost-effective:

(1) Off-the-shelf availability: Unlike CAR-T cells, which are patient-specific and require a lengthy and costly manufacturing process, CAR-NK cells can be derived from healthy donors and stored for immediate use [48]. This eliminates the need for individualized cell production and reduces the waiting time for patients.
(2) Safety: CAR-NK cells have been associated with fewer severe side effects such as cytokine release syndrome (CRS), neurotoxicity, and graft-versus-host disease (GVHD) [49]. This could potentially lead to lower healthcare costs related to the management of these side effects.
(3) Natural cytotoxicity: CAR-NK cells preserve the natural cytotoxicity of NK cells even if the expression of targeted tumor antigens is downregulated [48]. This could potentially lead to more effective treatment outcomes and, therefore, cost savings in the long run.
(4) Manufacturing costs: While the exact cost of manufacturing CAR-NK cells is not specified, the cost of manufacturing CAR-T cells has been estimated to be between USD 48,000 and USD 106,000 per dose [50]. The ability to produce CAR-NK cells from healthy donors and in larger batches could potentially reduce these costs.

In conclusion, while both CAR-T and CAR-NK cells have shown promise in cancer therapy, the unique characteristics of CAR-NK cells, including their off-the-shelf availability, safety profile, and natural cytotoxicity, make them potentially more cost-effective. However, more research is needed to fully understand the cost-effectiveness of CAR-NK cells in comparison to CAR-T cells (Table 1).

## 4. Development of CAR-NK Cells

### 4.1. CAR-NK Cell Preparation Process

The manufacturing of CAR-NK cells is a complex, multistage endeavor, carefully designed to optimize their therapeutic safety, effectiveness, and uniformity. The initiation of this process requires the acquisition of initial biological materials, which may encompass peripheral blood mononuclear cells (PBMCs) obtained from donors, umbilical cord blood, or iPSCs [51].

In the generation of PBMC-derived CAR-NK cells, leukapheresis serves as the initial step to procure PBMCs, which is followed by the selective removal of CD3+ T cells and enrichment of CD56+ NK cells [52]. These cells are then primed for activation with cytokines such as IL-2, or a synergistic cytokine blend including IL-2, IL-15, and IL-18. Subsequently, they are cultured alongside feeder cells over a span of 10 to 15 days to achieve full activation [53]. Prior to infusion into the patient, a lymphodepleting regimen is administered, and the support of IL-2 post-infusion is typically provided to promote CAR-NK cell persistence and activity [54]. In contrast, the production of CAR-NK cells from cord blood necessitates the initial isolation and amplification of CD34+ hematopoietic progenitor cells [55]. These progenitors are then guided through a differentiation process into functional NK cells by a milieu of cytokines including SCF, IL-7, IL-15, and IL-1, amongst others. This differentiation phase is completed after an extended culture period with feeder cells, lasting beyond 21 days [56].

**Figure 4:**
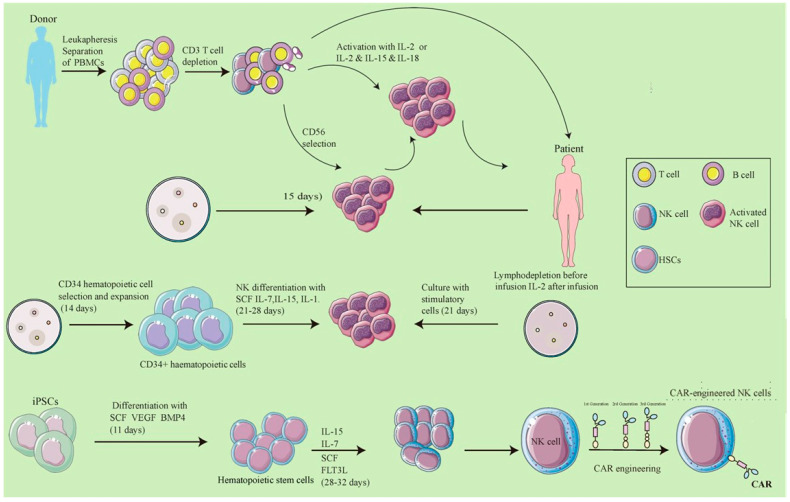
Production process of CAR-NK cells from various starting materials. The workflow begins with leukapheresis for PBMC collection, followed by CD3+ T cell depletion and activation with IL-2, IL-15, and IL-18. NK cells are isolated, cultured, and expanded. CD34+ cells are selected, expanded, and differentiated into NK cells using SCF, IL-7, and IL-15. The process concludes with cell culturing and patient infusion preparation, including lymphodepletion and IL-2 administration. An alternative pathway for CAR-NK cell derivation from iPSCs involves growth factors like SCF, VEGF, and BMP4 [14].

For CAR-NK cells derived from iPSCs, the protocol entails the progressive differentiation of iPSCs into hematopoietic progenitor cells through the action of SCF, vascular endothelial growth factor (VEGF), and bone morphogenetic protein 4 (BMP4) [57]. Following this, the progenitors are differentiated into NK cells using a tailored cocktail of cytokines, such as IL-15, IL-3, IL-7, SCF, and FLT3L [58]. The final maturation and expansion of these NK cells demand a 28-to-32-day cultivation with feeder cells [59]. Each of these methodologies underscores the nuanced and meticulous nature of CAR-NK cell production, a testament to the cutting-edge advances in cellular engineering that are setting new horizons in cancer immunotherapy (Figure 4).

### 4.2. Technological Advances and Innovations

CAR-NK cells represent a groundbreaking approach in immunotherapy, utilizing genetically engineered NK cells equipped with CARs [33]. These synthetic receptors are adept at recognizing specific tumor cell antigens, triggering the NK cells to annihilate the cancerous cells. A significant advantage of CAR-NK cells over their counterparts, CAR-T cells, lies in their derivation from allogeneic sources, like umbilical cord blood or induced pluripotent stem cells. This attribute negates the need for patient-specific manufacturing processes [6]. Moreover, CAR-NK cells present a substantially reduced risk of cytokine release syndrome and graft-versus-host disease, two severe complications often associated with CAR-T cell therapies [60].

The evolution of CAR-NK cell technology has been propelled by remarkable advancements in gene editing, cell engineering, and the field of cancer immunology. Pivotal developments include:

(1) Enhanced CAR Designs: The initial generation of CARs featured an scFv from an antibody targeting a tumor antigen, linked to a CD3ζ signaling domain for NK cell activation [61]. However, limitations in efficacy and persistence arose, with CAR-NK cells becoming exhausted or anergic after repeated activations. Addressing these drawbacks, subsequent generations of CARs now incorporate additional co-stimulatory domains, like CD28, 4-1BB, or OX40 [62]. These modifications significantly bolster the survival, proliferation, and functionality of CAR-NK cells [63]. Further, innovations have led to the exploration of diverse antigen recognition formats, such as NKG2D receptors, bispecific antibodies, or nanobodies, enhancing the specificity and adaptability of CAR-NK cells [64].
(2) Identification of Novel Therapeutic Targets: The efficacy and safety of CAR-NK cell therapy heavily depend on the chosen tumor antigens. Ideal targets are those predominantly expressed on tumor cells, minimizing off-target effects on healthy tissues [65]. However, this is challenging since many tumor antigens also appear on normal cells at lower levels. To circumvent this, researchers have discovered more tumor-specific targets. Some targets for genetic engineering in NK cells are uniquely expressed in tumor-specific conditions, like Hypoxia-inducible factor 1 in hypoxic environments [66], while others, like CD38 or CD19, exhibit distinct glycosylation patterns in tumor cells compared to their normal counterparts [67].
(3) Synergistic Combination Therapies: The integration of CAR-NK cells with other treatment modalities—such as chemotherapy, radiotherapy, checkpoint inhibitors, or cytokines—offers a promising strategy to elevate their effectiveness and safety [68]. These combinations can synergistically augment tumor infiltration, activation, persistence, and anti-tumor activity of CAR-NK cells [69]. For instance, combining CAR-NK cells with IL-15 or IL-21 can significantly enhance NK cell survival and functionality [70]. Additionally, pairing CAR-NK cells with PD-1 or PD-L1 inhibitors can disrupt the immune checkpoints that typically inhibit NK cell activity [71].

### 4.3. Strategies to Enhance CAR-NK Cells

One of the challenges in developing effective CAR-NK cell therapies is to overcome the tumor microenvironment, which can suppress the function and persistence of CAR-NK cells. Several strategies have been proposed to enhance the anti-tumor activity of CAR-NK cells, such as the use of specific cytokines, improving cell penetration abilities, and extending cell survival [72].

Cytokines are important mediators of immune responses and can modulate the proliferation, differentiation, activation, and survival of CAR-NK cells [6]. For example, IL-15 is a key cytokine for NK cell development and maintenance and has been shown to enhance the anti-tumor efficacy of CAR-NK cells in preclinical models [73]. IL-15 can also induce the expression of NKG2D, a natural cytotoxicity receptor that can recognize stress-induced ligands on tumor cells [74]. Other cytokines that have been explored for CAR-NK cell therapy include IL-2, IL-7, IL-12, IL-18, and IL-21 [75].

Another strategy to improve the anti-tumor activity of CAR-NK cells is to enhance their ability to penetrate solid tumors, which are often characterized by high interstitial pressure, a dense extracellular matrix, and low oxygen levels [76]. One approach is to engineer CAR-NK cells with chemokine receptors that can guide them to the tumor site, such as CXCR2 [77,78] or CCR2 [79]. Another approach is to co-express enzymes that can degrade the extracellular matrix, such as hyaluronidase or matrix metalloproteinases [68]. A third approach is to co-express hypoxia-inducible factors that can increase the expression of glycolytic enzymes and angiogenic factors, which can help CAR-NK cells adapt to the hypoxic tumor microenvironment [80].

A third strategy to enhance the anti-tumor activity of CAR-NK cells is to extend their survival and persistence in vivo, which can be affected by factors such as immunosuppressive cells, soluble factors, and metabolic stress [81]. One way to increase the survival of CAR-NK cells is to co-express anti-apoptotic genes, such as Bcl-2 or Bcl-xL [82]. Another way is to co-express immune checkpoint inhibitors, such as PD-1 or CTLA-4, which can prevent the exhaustion and dysfunction of CAR-NK cells [83]. A third way is to co-express metabolic regulators, such as AMPK or PGC-1α, which can enhance the mitochondrial function and oxidative phosphorylation of CAR-NK cells [84].

In the dynamic field of CAR-NK cell therapy, a multitude of cutting-edge strategies centered around novel cytokines and receptors are being pursued to surmount various challenges, including tumor heterogeneity, antigen escape, and the immunosuppressive tumor microenvironment [13]. One such innovative strategy involves leveraging NK cells as a source for CAR-NK cells [85], offering a route to enhance their activation and cytotoxic capabilities against tumor cells. Equally significant is the exploration of the cytokine-inducible SH2-containing protein (CIS) as a regulatory mechanism in CAR-NK cell signaling [86]. CIS acts as a negative feedback regulator, aiming to finely tune the activation levels of CAR-NK cells, which could be crucial in preventing overactivation and reducing the risk of cytokine release syndrome, a common complication in cell-based therapies [87].

Further enhancing the CAR-NK cell repertoire, researchers are investigating the application of IL-15 super agonist complexes [70], which hold the potential to significantly boost the survival, persistence, and anti-tumor efficacy of CAR-NK cells. In parallel, IL-21 is being harnessed to promote the expansion and differentiation of CAR-NK cells [88], aiming to generate a more potent and diverse NK cell population capable of effectively targeting a broader spectrum of tumor antigens. Additionally, IL-12 is garnering attention for its ability to augment the anti-tumor activity of CAR-NK cells and aid in the formation of long-lasting immunological memory, an essential feature for sustained cancer remission [89].

Another groundbreaking approach involves the development of bispecific antibodies for cytokine receptors [90]. These receptors are designed to broaden the specificity and versatility of CAR-NK cells, enabling them to target multiple tumor antigens simultaneously and respond more effectively to various signals within the tumor microenvironment [91]. This strategy could be pivotal in enhancing the targeting accuracy of CAR-NK cells, potentially overcoming one of the major hurdles in cancer immunotherapy—the adaptability of tumors [11].

**Figure 5:**
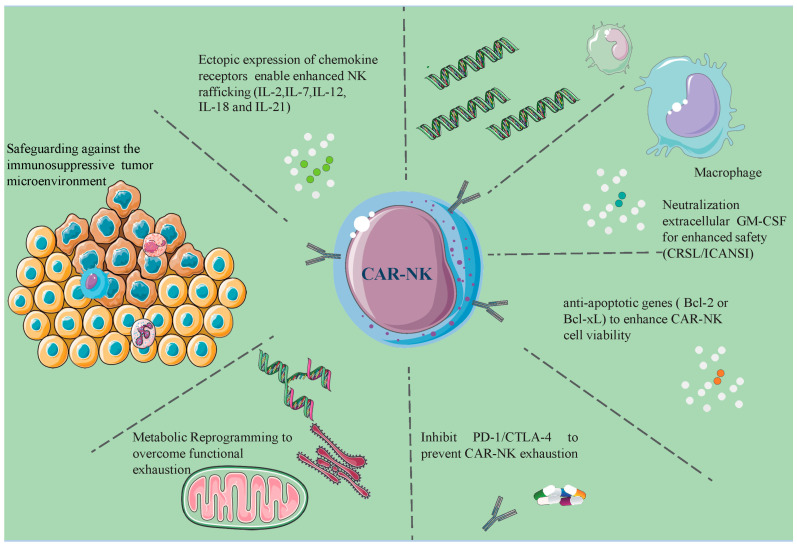
Multifaceted mechanisms of CAR-NK cell therapy in the tumor microenvironment. This illustration demonstrates key advancements such as genetic disruption of immune checkpoints in monocytes, ectopic expression of chemokine receptors for improved NK cell trafficking, and neutralization of extracellular GM-CSF to increase safety. Also depicted are metabolic reprogramming techniques to prevent functional exhaustion, cytokine preconditioning for NK cell memory enhancement, and the targeted ablation of endogenous NK cell proteins to optimize combination therapy efficacy [92].

Together, these advancements represent a significant leap forward in the quest to enhance the efficacy, durability, and safety of CAR-NK cell therapies. By addressing the current limitations and harnessing the power of innovative cytokines and receptor technologies, these strategies are laying the groundwork for more effective and tailored cancer treatments, offering hope to patients battling various malignancies (Figure 5).

### 4.4. Synthetic Biology Applications in CAR-NK Cells

Synthetic biology, a cross-disciplinary field blending engineering principles with biological systems, plays a pivotal role in advancing CAR-NK cell therapies [93]. In this innovative approach, CAR-NK cells are equipped with synthetic receptors that recognize and target specific antigens on tumor cells, initiating the NK cell’s cytotoxic response. Despite their potential, CAR-NK cells face challenges such as target antigen selection, CAR expression regulation, enhancement of cell persistence and homing, and mitigation of off-target effects and immunogenicity [94]. To address these challenges, synthetic biology offers a suite of tools and strategies, particularly in modifying cytokine signaling pathways, crucial for the survival, proliferation, activation, and function of CAR-NK cells [95].

One notable application is the engineering of CAR-NK cells to express IL-15 or its receptor IL-15Rα constitutively, leveraging IL-15’s role in NK cell development and maintenance. This modification aims to enhance CAR-NK cell persistence and antitumor activity in vivo [6,61]. Additionally, synthetic biology techniques are employed to create CAR-NK cells that secrete IL-12 upon antigen recognition. IL-12, a pro-inflammatory cytokine, boosts NK cell activation and cytotoxicity, thereby enhancing antitumor efficacy and countering immunosuppression in the tumor microenvironment [96]. Furthermore, to prevent NK cell exhaustion and enhance sensitivity to tumor antigens, CAR-NK cells are being engineered to lack PD-1 expression or incorporate a dominant-negative PD-1 mutant [97]. This manipulation addresses the inhibitory effects of the PD-1 pathway on NK cell function [98]. These examples illustrate some of the potential applications of synthetic biology in CAR-NK cell therapy. With its ability to provide precise and versatile modifications, synthetic biology stands as a powerful platform for designing more effective CAR-NK cell therapies, offering new avenues for cancer treatment.

## 5. Clinical Applications and Future Outlook

### 5.1. Clinical Trials of CAR-NK Cells:

CAR-NK cells are a promising immunotherapy strategy for the treatment of various cancers [14]. Several clinical trials have been conducted or are ongoing to evaluate the safety and efficacy of CAR-NK cells in different tumor types. Here, we provide an overview of some of these trials and their preliminary results. The first clinical trial of CAR-NK cells was reported by Liu et al. in 2020 [6]. They used allogeneic NK cells derived from cord blood and transduced them with a retroviral vector encoding a CD19-specific CAR. The CAR-NK cells were infused into 25 patients with relapsed or refractory B-cell malignancies, including non-Hodgkin lymphoma (NHL), chronic lymphocytic leukemia (CLL), and acute lymphoblastic leukemia (ALL) [6]. The results showed that the CAR-NK cells were well tolerated, with no cases of cytokine release syndrome (CRS) or GVHD. The overall response rate (ORR) was 68%, with 12 patients achieving complete remission (CR) and 5 achieving partial remission (PR). The median progression-free survival (PFS) was 13.8 months, and the median overall survival (OS) was not reached.

In a recent Phase I/II trial (NCT03056339), researchers evaluated cord-blood-derived CAR-NK cell therapy targeting CD19 in 37 patients with relapsed or refractory B-cell malignancies. The trial demonstrated a favorable safety profile, with no severe cytokine release syndrome, neurotoxicity, or GVHD observed. At 100 days post-treatment, the overall response rate (OR) was 48.6%. Notably, patients who achieved an OR had higher levels and longer persistence of CAR-NK cells. The study emphasized the importance of donor selection, with optimal cord blood units (CBUs) cryopreserved within 24 h of collection yielding better outcomes. CAR/IL-15 NK cells from these optimal CBUs showed superior antitumor activity in mouse models [99].

Other ongoing clinical trials of CAR-NK cells include NCT03692637, which is testing a CD19-specific CAR-NK cell product derived from iPSCs in patients with B-cell malignancies (ClinicalTrials.gov Identifier: NCT03692637); NCT04324996, which is testing a CD30-specific CAR-NK cell product derived from PBMCs in patients with Hodgkin lymphoma or anaplastic large cell lymphoma (ClinicalTrials.gov Identifier: NCT04324996); and NCT04613952, which is testing a mesothelin-specific CAR-NK cell product derived from PBMCs in patients with malignant pleural mesothelioma (ClinicalTrials.gov Identifier: NCT04613952). These trials are expected to provide more evidence on the feasibility, safety, and efficacy of CAR-NK cells in various cancer settings.

### 5.2. Future Research Directions and Challenges

CAR-NK cell therapy, with its significant potential in cancer treatment, particularly in hematological malignancies, faces several challenges and areas for future research to enhance its efficacy and safety. Some pivotal directions and challenges include the following:

(1) Optimizing CAR Design and Engineering:

A key area of focus is the optimization of CAR constructs to improve their specificity, affinity, and functionality. This could involve developing bispecific or multispecific CARs capable of targeting multiple tumor antigens, which would be particularly useful in treating cancers with high antigenic variability [100]. Incorporating costimulatory domains or cytokines into CAR designs is another strategy to modulate CAR-NK cell activation and survival, potentially improving their persistence and reducing toxicity [101]. Additionally, the integration of suicide genes or switchable receptors offers a method to control the longevity and potential adverse effects of CAR-NK cells in vivo, providing a safety switch to mitigate risks [102].

(2) Developing Novel Sources and Production Methods:

The search for new sources and methods for generating CAR-NK cells is crucial. Utilizing iPSCs or umbilical cord blood (UCB) as alternative NK cell sources opens possibilities for a more readily available and versatile cell supply [103]. Advances in gene delivery, using either viral or non-viral vectors, and innovative ex vivo expansion or in vivo proliferation techniques are essential to produce CAR-NK cells in sufficient quantities and qualities [104].

(3) Overcoming Tumor Microenvironment Barriers:

The tumor microenvironment (TME) presents significant barriers that can impair the function and homing of CAR-NK cells [105]. Combination therapies that include immune checkpoint inhibitors, cytokines, or chemotherapeutic agents could help modulate the immunosuppressive nature of the TME [106]. Additionally, employing nanoparticles or antibodies to enhance CAR-NK cell delivery and penetration into solid tumors, along with using advanced imaging techniques or biomarkers to track their distribution and effectiveness, are promising approaches to overcome these challenges [107,108].

(4) Addressing Off-Target Effects and Adverse Events:

The potential for off-target effects and adverse events remains a concern in CAR-NK cell therapy. Research focused on antigen-specific or antigen-loss relapse models is vital for evaluating risks associated with on-target/off-tumor toxicity [13]. Preclinical animal models and clinical trials are essential for assessing the safety and feasibility of CAR-NK cell therapy across different cancer types and stages [109]. Additionally, the development and standardization of protocols and guidelines for managing adverse events such as CRS or neurotoxicity are crucial for the safe implementation of this therapy [110].

(5) Enhancing Surgical Oncology Outcomes through CAR-NK Cell Therapy Integration

In the evolving landscape of oncological treatment, the advent of CAR-NK cell therapy presents a promising synergy with surgical interventions, particularly in the multimodal management of cancer [111]. The strategic integration of CAR-NK cells could enhance the efficacy of surgical resection and provide a comprehensive approach to cancer care.

Preoperative CAR-NK Cell Therapy: Prior to surgical intervention, CAR-NK cell therapy could be deployed to reduce tumor burden. The cytotoxic activity of CAR-NK cells against tumor cells can potentially decrease tumor size, rendering previously inoperable tumors amenable to surgery [14]. Additionally, this reduction in tumor mass may lead to less extensive surgeries, diminishing the risk of postoperative complications and preserving more of the patient’s healthy tissue [68].

Adjuvant CAR-NK Cell Therapy: Post-surgical application of CAR-NK cells could play a pivotal role in eradicating residual malignant cells that evade surgical excision [112]. This could be particularly beneficial in the context of microscopic residual disease, which often contributes to cancer recurrence. Administering CAR-NK cell therapy following surgery may decrease the likelihood of relapse, offering a proactive approach to sustain the disease-free interval [113].

Combination Therapy for Comprehensive Care: The incorporation of CAR-NK cell therapy into the broader oncological treatment paradigm, alongside surgical resection, radiation, and chemotherapy, reflects a comprehensive strategy that could maximize therapeutic outcomes [114]. By providing a targeted immunotherapeutic intervention, CAR-NK cells can complement the direct physical removal of tumor mass, potentially preventing local and systemic relapse, a key concern in surgical oncology [115].

By harnessing the specificity and cytotoxic potential of CAR-NK cells, we can envision a collaborative model where immunotherapy and surgery work in concert to tackle the complex nature of cancer. This approach aligns with the journal’s commitment to fostering innovations that support the surgeon’s role in cancer care, advocating for a holistic and patient-centered approach to treatment.

(6) Ethical Considerations in CAR-NK Cell Therapy:

In advancing CAR-NK cell therapy, addressing ethical challenges is paramount for patient safety, public trust, and equitable treatment access [116]. Essential considerations include obtaining informed consent, where patients must be thoroughly educated about the therapy’s complexities, potential risks, and long-term implications to ensure informed decisions [13]. Additionally, the genetic modifications central to CAR-NK therapy heighten privacy concerns, necessitating robust measures to protect sensitive patient data and maintain confidentiality [81]. Beyond the clinical trial phase, post-trial care and monitoring are crucial for assessing long-term effects and ensuring the ongoing welfare of patients [13]. Together, these ethical practices underscore the commitment to patient autonomy, privacy, and sustained care, forming the backbone of responsible CAR-NK cell therapy deployment.

## 6. Summary

CAR-NK cells, derived from various sources like peripheral blood, umbilical cord blood, and induced pluripotent stem cells, offer a promising alternative due to their inherent advantages such as reduced risk of cytokine release syndrome and the ability to target both hematological malignancies and solid tumors without the need for human leukocyte antigen (HLA) matching. As CAR-NK cells have shown efficacy and safety in treating hematological malignancies, with ongoing research into their use for solid tumors, refining their design, manufacturing processes, and delivery mechanisms remains a priority. The integration of advancements in synthetic biology, gene editing, and immunology holds promise for enhancing the effectiveness of CAR-NK cell therapies. Ethical considerations, including patient autonomy and privacy, are paramount as we navigate the complexities of this evolving field, ensuring that the new wave of immunotherapy not only achieves clinical success but also aligns with broader health economic and ethical standards. Thus, CAR-NK cell therapy represents a significant shift in cancer immunotherapy, with the potential to redefine oncological care and offer new avenues for treatment.

In conclusion, CAR-NK cell therapy emerges as a promising and innovative approach in the realm of immunotherapy, offering a compelling alternative to CAR-T cells with its potential for reduced toxicity and broader applicability. Given the current stage of CAR-NK cell therapy, which has not yet been widely applied in clinical settings, it is premature to conclusively assess its cost-effectiveness compared to CAR-T cell therapies. The lack of large-scale clinical application limits the availability of comprehensive economic data, highlighting the need for future research to conduct detailed cost analyses. Such studies are essential to understand the economic implications fully and ensure the sustainability and accessibility of these therapies as they progress toward broader clinical adoption.

## Figures and Tables

**Table 1:** Comparison between CAR-T and CAR-NK Cells.

Parameter	CAR-T Cells	CAR-NK Cells
Intracellular Signaling Domains	CD3ζ with co-stimulatory domains (CD28 and 4-1BB)	NK-specific signaling domains (2B4, DAP10, DAP12).
Cell Source	Autologous MHC-matched allogeneic	Autologous and non-MHC-matched allogeneic cell lines, Cord blood and iPSCs
Off-the-shelf Ready-to-use CAR Product	autologous, or MHC-matched allogeneic CAR-T cells	NK cell lines, Allogeneic NK cells possible (poor recovery rate)
In Vitro Expansion Capability	Yes	Yes
Cell Killing Mechanism	CAR-dependent cellular cytotoxicity	NK cell-mediated cytotoxicity
Cytokine Release Syndrome and Neurotoxicity	Common and usually severe	Less common
